# Prognostic value of platelet-to-lymphocyte ratio in patients with unresectable hepatocellular carcinoma undergoing transarterial chemoembolization and tyrosine kinase inhibitors plus immune checkpoints inhibitors

**DOI:** 10.3389/fonc.2024.1293680

**Published:** 2024-01-23

**Authors:** Yiwan Guo, Wenlong Wu, Bo Sun, Tingting Guo, Keke Si, Chuansheng Zheng, Xin Li

**Affiliations:** ^1^ Department of Radiology, Union Hospital, Tongji Medical College, Huazhong University of Science and Technology, Wuhan, China; ^2^ Department of Interventional Radiology, Union Hospital, Tongji Medical College, Huazhong University of Science and Technology, Wuhan, China

**Keywords:** platelet-to-lymphocyte ratio, hepatocellular carcinoma, transarterial chemoembolization, tailored tyrosine kinase inhibitors, immune checkpoint inhibitors

## Abstract

**Purpose:**

To investigate the prognostic value of platelet-to-lymphocyte ratio (PLR) in patients with unresectable hepatocellular carcinoma (uHCC) treated with transarterial chemoembolization (TACE) and tailored tyrosine kinase inhibitors (TKIs) plus immune checkpoints inhibitors (ICIs).

**Materials and methods:**

Ninety-eight patients from May 2018 to January 2022 in our hospital were enrolled in this study. The receiver operating characteristic (ROC) curve analysis was performed and the corresponding Youden index was used to determine the optimal PLR cut-off. Overall survival (OS), progression-free survival (PFS), and adverse events (AEs) of patients were evaluated based on the PLR cut-off. The factors affecting survival were assessed using univariate and multivariate Cox proportional hazards regression analyses.

**Results:**

The PLR cut-off was 98.89. There were 49 patients in the low pretreatment PLR group (PLR ≤ 98.89) and 49 patients in the high PLR group (PLR > 98.89). Patients with low pretreatment PLR had significantly longer median OS (25.7 months vs 16.1 months; P < 0.001) and PFS (14.9 months vs 10.2 months; P < 0.001) than those with high pretreatment PLR. The multivariate analysis revealed that ALT, tumor size, and PLR are risk factors affecting OS. The three independent factors affecting PFS are tumor size, AFP, and PLR. The AEs were tolerable and manageable.

**Conclusion:**

The low pretreatment PLR (PLR ≤ 98.89) was an independent protective factor for the survival outcomes of patients in this study. PLR was helpful for clinicians to predict the prognosis and identify the patients with uHCC who were most likely to benefit from TACE + TKIs + ICIs.

## Introduction

Hepatocellular carcinoma (HCC) is the third leading cause of cancer-related death in the world (1). Patients who are diagnosed with early-stage HCC have the opportunity to undergo curative treatments (2, 3). Since the onset of HCC is insidious, a majority of patients with HCC are diagnosed at intermediate or advanced stage and are not suitable for curative resection (4).

According to the guidelines (5, 6), transarterial chemoembolization (TACE) has been recommended as a standard treatment for intermediate and advanced HCC. Since the efficacy of TACE is associated with tumor size, vascular invasion and distant metastasis (7), it is challenging to achieve complete tumor necrosis using TACE alone. In addition, TACE could increase the expression of programmed cell death ligand 1 (PD-L1) and vascular endothelial growth factors (VEGF) as a result of the hypoxic microenvironment after embolization, contributing to the tumor recurrence and metastasis (8, 9).

It is known that immune checkpoints, including programed cell death protein 1 (PD-1), programed cell death ligand 1 (PD-L1) and cytotoxic T-lymphocyte-associated protein 4 (CTLA-4), can suppress the T-cell-mediated immune responses, which permits cancer cells to escape from the immune destruction (10). Immune checkpoint inhibitors (ICIs) such as camrelizumab and atezolizumab act to block the interaction of immune checkpoints and the corresponding ligands. As a result, tumor-reactive T cells are able to overcome the negatively regulatory mechanisms caused by immune checkpoints and facilitate an effective anti-tumor response (11).

Angiogenic factors such as VEGF can bind to VEGF receptors (VEGFRs) to suppress immune responses by inducing vascular abnormalities, inhibiting antigen presentation, or enhancing the activity of regulatory T cells to suppress the immune system (12, 13). Tyrosine kinase inhibitors (TKIs) can exactly block the intracellular domain of VEGFR to impede the immunosuppression effects of VEGF (14).

Thus, systemic therapy, including ICIs and TKIs, has been recommended as the first-line treatment for patients with advanced HCC (15). Based on the guidelines for primary liver cancer (16), it is recommended to combine TACE with systemic therapy to enhance the efficacy of TACE. And many studies have investigated the efficacy of TACE and TKIs plus ICIs, demonstrating significantly higher tumor response and survival benefits (17–19).

Some studies have shown that inflammatory and immune environments play an important role in the formation and progression of HCC (20, 21). And many studies have evaluated the effects of various inflammatory and immune biomarkers in predicting the outcomes of patients with malignant tumors (22–24). High platelet counts can stimulate angiogenesis and tumor proliferation by enhancing the secretion of growth factors, such as VEGF and platelet-derived growth factors (25). Decreased lymphocyte counts are related to an insufficient immunologic reaction to the tumor, which consequently enable tumor progression and metastasis (26). Increased platelet counts along with decreased lymphocyte counts lead to an elevated PLR, which is associated with unfavorable clinical outcome in HCC patients receiving TACE alone or TACE plus TKIs (27–29). However, the prognostic value of PLR for uHCC patients treated with TACE + TKIs + ICIs has not been evaluated.

This study aimed to investigate the effectiveness of pretreatment PLR in predicting the survival outcomes of uHCC patients treated with TACE + TKIs + ICIs.

## Materials and methods

### Patients

The Institutional Review Board in our hospital approved this retrospective study, and the informed consent was waived. This study was conducted in accordance with the Declaration of Helsinki.

Patients with uHCC received TACE + TKIs + ICIs between May 2018 and January 2022 in our hospital were enrolled in this study. HCC was diagnosed by pathological examination or noninvasive criteria based on the European Association for the Study of the Liver (EASL) guidelines (6). A multidisciplinary team determined the patients’ treatment decisions.

The inclusion criteria for this study were as follows: 1) age ≥ 18 years; 2) confirmed diagnosis with uHCC; 3) Eastern Cooperative Oncology Group (ECOG) scores ≤ 1; 4) Child-Pugh A or B; 5) adequate cardiac, renal and coagulation function; 6) treated with TACE + TKIs + ICIs.

The exclusion criteria were as follows: 1) previous HCC-related treatments, including hepatic resection, liver transplantation, systemic therapy, local ablation or TACE; 2) Child-Pugh C; 3) presence of other malignancies in addition to HCC; 4) incomplete data.

### Treatment protocol

TACE was performed under local anesthesia via right femoral artery. The Seldinger technique and angiography were performed to identify the tumor-feeding arteries and assess the tumor burden. According to the tumor burden, 5-15mL of emulsion containing 10-20 mg of doxorubicin hydrochloride (Hisun Pharmaceutical Co.LTD, Zhejiang, China) was mixed with 5-10 mL of lipiodol (Lipiodol Ultrafluido, Guerbet, France) and injected into the tumor-feeding arteries through a 3-F microcatheter. Finally, an appropriate amount of gelatin sponge particles (350-560 µm; Cook) was injected into the tumor-feeding arteries to induce embolization.

TKIs including sorafenib (800 mg), lenvatinib (8 or 12 mg), and apatinib (500 mg) were administered orally daily. ICI immunotherapy with intravenous fixed-dose camrelizumab (200 mg) was performed every 3 weeks until disease progression or unexpected toxicity was observed. The dose and interval of TKIs were adjusted according to the toxicity and disease conditions. The administration of TKIs and ICIs should be stopped when unacceptable toxicity occurred or no clinical benefits were observed. What’s more, TKIs and ICIs were discontinued for 3 days before and after TACE.

### Outcomes and follow-up

All laboratory indicators and radiological data were collected within 7 days of initial treatment. PLR was calculated as absolute platelet count divided by absolute lymphocyte count prior to the initial treatment. All patients were followed up every 4-8 weeks. The laboratory and imaging information of patients were recorded at each appointment. Two radiologists with more than 10 years experience in abdominal radiology evaluated the imaging examinations. Both of them were blinded to the patients’ clinical information. TACE was recommended if the patient had a residual tumor or disease progression during the follow-up. Adverse events (AEs) in this study were monitored and recorded by experienced nurses according to the Common Terminology Criteria for Adverse Events (CTCAE) version 5.0 (30).

Overall Survival (OS) and Progression-Free Survival (PFS) were outcomes of this study. OS was defined as the interval from the initial treatment to death or the last follow-up. PFS was defined as the time between the initial treatment and disease progression according to the modified Response Evaluation Criteria in Solid Tumors (mRECIST) (31), death or the last follow-up.

### Statistical analysis

SPSS version 24.0 (IBM, Chicago, Illinois, USA) was used for the statistical analyses. Continuous variables and categorical variables were presented as median (interquartile range) and frequencies (percentages), respectively. The time-dependent ROC curve analysis was performed and the corresponding Youden index was used to determine the optimal PLR cut-off for patients with uHCC. Continuous variables at baseline for the high and low PLR groups were compared using Student’s t-test or Mann-Whitney U test. Chi-square test or Fisher’s exact test was used to compare categorical variables. OS and PFS curves were drawn by the Kaplan-Meier method and were compared using log-rank tests. Risk factors related to OS and PFS were identified by univariate and multivariate Cox proportional hazards regression analysis. Factors with P < 0.05 at univariate analysis were included in the multivariate analysis. P < 0.05 was considered statistically significant.

## Results

### Baseline statistics

**Figure 1:**
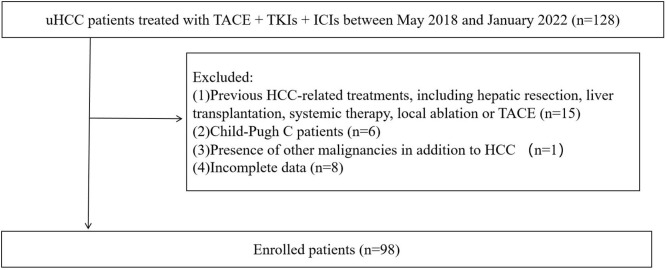
Flow chart.

**Figure 2:**
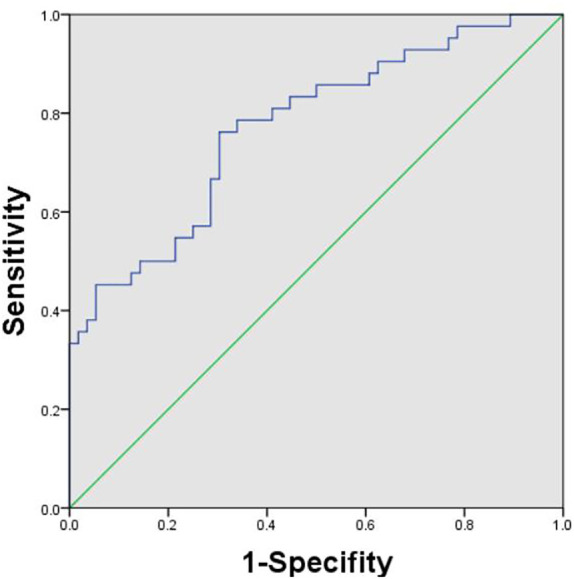
Receiver operating characteristic curve analysis was performed to determine the optimal cut-off for PLR. The cut-off was 98.89. PLR, platelet-to-lymphocyte ratio.

During the follow-up, a total of 128 uHCC patients treated with TACE + TKIs + ICIs were enrolled in this study. However, thirty patients were excluded according to the exclusion criteria (**Figure 1**). The ROC curve analysis was performed and the Youden index suggested that the optimal PLR cut-off was 98.89. The area under the ROC (AUC) curve was 0.77 (**Figure 2**). According to the cut-off, forty-nine (50.0%) patients with PLR > 98.89 were divided into the high PLR group and the rest 49 (50.0%) patients with PLR ≤ 98.89 were divided into the low PLR group. The baseline characteristics of these patients were presented in **Table 1**, with no statistical difference between the two groups.

**Table 1:** The baseline characteristics of patients.

Characteristics	PLR ≤ 98.89	PLR >98.89	P value
**Age(years)**	57.2 ± 6.54	56.8 ± 5.10	0.693
**ALT**	33.1 ± 16.6	30.2 ± 16.7	0.384
**AST**	38.9 ± 17.3	43.2 ± 15.1	0.189
**TB (µmol/L)**	16.9 ± 5.77	16.6 ± 5.2	0.808
**Albumin(g/dl)**	36.2 ± 5.0	35.4 ± 5.8	0.469
**PLT**	136.8 ± 59.2	140.34 ± 63.7	0.382
**Tumor size (cm)**	7.1 ± 3.7	8.0 ± 4.5	0.251
**Sex**			0.493
male	44	42	
female	5	7	
Treatment protocols			0.670
TACE + sorafenib + camrelizumab	22	24	
TACE + lenvatinib + camrelizumab	17	13	
TACE + apatinib + camrelizumab	10	12	
**Number of tumors**			0.686
1	27	24	
≥2	22	25	
**BCLC stage**			0.211
B	22	15	
C	27	34	
**Cirrhosis**			1.000
Yes	47	48	
No	2	1	
**ascites**			0.289
Yes	14	20	
No	35	29	
**Portal vein invasion**			0.225
Yes	20	27	
No	29	22	
**Extrahepatic metastases**			0.200
Yes	24	32	0.153
No	25	17	
**AFP (ng/ml)**			0.538
<400	18	22	
≥400	31	27	
**Child-Pugh**			0.815
A	36	38	
B	13	11	
**ECOG performance**			0.076
0	39	30	
1	10	19	

ALT, alanine aminotransferase; AST, aspartate aminotransferase; TB, total bilirubin; PLT, platelet; BCLC, Barcelona Clinic Liver Cancer; AFP, a-fetoprotein; ECOG, Eastern Cooperative Oncology Group.

### OS and PFS

**Figure 3:**
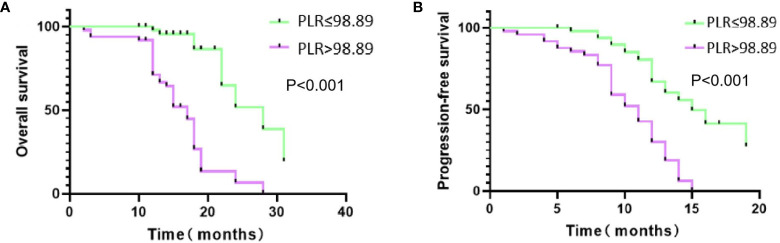
Kaplan-Meire (K-M) curves of two groups. **(A)** K-M curve for overall survival (OS); **(B)** K-M curve for progression-free survival (PFS).

The Kaplan–Meier analysis showed that the median OS (mOS) of patients in the low PLR group was higher than that of those in the high PLR group (25.7 months vs 16.1 months; P < 0.001) (**Figure 3A**). Similarly, the median PFS of patients in the low PLR group was also higher than the high PLR group (14.9 months vs 10.2 months; P < 0.001) (**Figure 3B**).

### Risk factors affecting OS and PFS

Univariate Cox proportional hazards regression analysis revealed that alanine transaminase (ALT) (hazard ratio [HR]: 1.035; 95% confidence interval [CI]: 1.002-1.069; P = 0.039), tumor size (HR: 1.186; 95% CI: 1.059-1.328; P = 0.003), and PLR (HR: 8.547; 95% CI: 2.902-25.170; P = 0.000) were risk factors affecting OS (**Table 2**). The factors related to PFS (**Table 3**) included tumor size (HR: 1.135; 95% CI: 1.031-1.249; P = 0.010), alpha-feto protein (AFP) (HR: 2.516; 95% CI: 1.281-4.940; P = 0.007), and PLR (HR: 4.882; 95% CI: 2.336-10.205; P = 0.000). Multivariate Cox proportional hazards regression analysis identified three risk factors affecting OS: ALT (HR: 1.022; 95% CI: 1.006-1.038; P = 0.006), tumor size (HR: 1.121; 95% CI: 1.045-1.202; P = 0.001), and PLR (HR: 6.680; 95% CI: 3.055-14.606; P = 0.000). Three independent factors affected PFS: tumor size (HR: 1.110; 95% CI: 1.037-1.188; P = 0.003), AFP (HR: 1.940; 95% CI: 1.095-3.437; P = 0.023) and PLR (HR: 3.540; 95% CI: 2.004-6.254; P = 0.000).

**Table 2:** Univariate and multivariate Cox proportional hazards regression analysis of risk factors for OS.

Varaiable	Univariate analysis		Multivariate analysis	
	HR (95%CI)	P value	HR (95%CI)	P value
**Age (years)**	0.970 (0.899-1.046)	0.429		
**ALT**	1.035 (1.002-1.069)	**0.039**	1.022 (1.006-1.038)	**0.006**
**AST**	0.985 (0.954-1.017)	0.356		
**TB**	1.004 (0.913-1.105)	0.929		
**Albumin**	0.919 (0.842-1.003)	0.058		
**PLT**	0.997 (0.987-1.007)	0.548		
**Tumor size (cm)**	1.186 (1.059-1.328)	**0.003**	1.121 (1.045-1.202)	**0.001**
**Sex**		0.535		
Male	1			
Female	0.667 (0.186-2.399)			
**Number of tumors**		0.609		
1	1			
≥2	0.777 (0.296-2.041)			
**BCLC stage**		0.501		
B	1			
C	1.864 (0.303-11.464)			
**cirrhosis**		0.323		
Yes	1			
No	0.294 (0.026-3.330)			
**ascites**		0.456		
Yes	1			
No	0.6148 (0.193-2.122)			
**Portal vein invasion**		0.187		
Yes	1			
No	3.206 (0.843-10.200)			
**Extrahepatic metastasis**		0.310		
Yes	1			
No	2.103 (0.501-8.823)			
**AFP**		0.560		
<400	1			
≥400	1.295 (0.543-3.087)			
**Child-Pugh**		0.198		
A	1			
B	3.355 (0.801-12.055)			
**ECOG performance**		0.692		
0	1			
1	1.177 (0.526-2.632)			
**PLR**		**0.000**		**0.000**
PLR ≤98.89	1		1	
PLR >98.89	8.547 (2.902-25.170)		6.680 (3.055-14.606)	

OS, overall survival; HR, hazard ratio; CI, confidence interval; ALT, alanine transaminase; AST, aspartate aminotransferase; TB, total bilirubin; PLT, platelet; BCLC, Barcelona Clinic Liver Cancer; AFP, a-fetoprotein; ECOG, Eastern Cooperative Oncology Group; PLR, platelet-to-lymphocyte ratio.

The bold values means P < 0.05, which is considered statistically significant.

**Table 3:** Univariate and multivariate Cox proportional hazards regression analysis of risk factors for PFS.

Varaiable	Univariate analysis		Multivariate analysis	
	HR (95%CI)	P value	HR (95%CI)	P value
**Age (years)**	1.007 (0.944-1.075)	0.828		
**ALT**	0.995 (0.967-1.022)	0.697		
**AST**	1.010 (0.984-1.036)	0.446		
**TB**	0.968 (0.886-1.058)	0.476		
**Albumin**	1.010 (0.916-1.115)	0.835		
**PLT**	0.996 (0.989-1.004)	0.370		
**Tumor size (cm)**	1.135 (1.031-1.249)	**0.010**	1.110 (1.037-1.188)	**0.003**
**Sex**		0.230		
Male	1			
Female	0.574 (0.232-1.420)			
**Number of tumors**		0.423		
1	1			
≥2	1.333 (0.6600-2.690)			
**BCLC stage**		0.567		
B	1			
C	1.461 (0.399-5.344)			
**cirrhosis**		0.078		
Yes	1			
No	0.268 (0.061-1.167)			
**ascites**		0.172		
Yes	1			
No	0.489 (0.175-1.366)			
**Portal vein invasion**		0.997		
Yes	1			
No	1.002 (0.342-2.937)			
**Extrahepatic metastasis**		0.662		
Yes	1			
No	0.662 (0.250-1.755)			
**AFP**		**0.007**		**0.023**
<400	1		1	
≥400	2.516 (1.281-4.940)		1.940 (1.095-3.437)	
**Child-Pugh**		0.963		
A	1			
B	0.969 (0.253-3.704)			
**ECOG performance**		0.864		
0	1			
1	1.063 (0.526-2.151)			
**PLR**		**0.000**		**0.000**
PLR ≤98.89	1		1	
PLR >98.89	4.882 (2.336-10.205)		3.540 (2.004-6.254)	

PFS, progression-free survival; HR, hazard ratio; CI, confidence interval; ALT, alanine transaminase; AST, aspartate aminotransferase; TB, total bilirubin; PLT, platelet; BCLC, Barcelona Clinic Liver Cancer; AFP, a-fetoprotein; ECOG, Eastern Cooperative Oncology Group; PLR, platelet-to-lymphocyte ratio.

The bold values means P < 0.05, which is considered statistically significant.

### Safety

All AEs were presented in **Table 4**. There was no treatment-related death observed in this study. The most common TACE-related AEs were postembolization syndrome that included nausea (57.2%), vomiting (34.7%), abdominal pain (59.2%), and fever (83.7%). And the most common drug-related AEs were hypertension (24.5%), fatigue (49.0%), headache (14.3%), skin capillary hyperplasia (18.4%), hypothyroidism (22.4%), and pneumonia (2.0%). For grade 3 or 4 AEs, only nausea, fever, hypertension and fatigue had incidences of >5%. (**Table 4**).

**Table 4:** Adverse events of two groups.

Adverse events	Any grade			Grade III or IV		
	PLR ≤ 98.89 (N,%)	PLR>98.89 (N,%)	P value	PLR ≤ 98.89 (N,%)	PLR>98.89 (N,%)	P value
**Nausea**	28(57.2%)	27 (55.1%)	0.839	4 (8.2%)	3 (6.1%)	0.696
**Vomiting**	17 (34.7%)	19 (38.8%)	0.675	1 (2.0%)	1 (2.0%)	1.000
**Abdominal pain**	29 (59.2%)	30 (61.2%)	0.836	1 (2.0%)	2 (4.1%)	0.560
**Fever**	41 (83.7%)	39 (79.6%)	0.602	7 (14.3%)	5 (10.2%)	0.513
**Hypertension**	12 (24.5%)	13 (26.5%)	0.817	3 (6.1%)	2 (4.1%)	0.648
**Fatigue**	24 (49.0%)	25 (51.0%)	0.840	3 (6.1%)	4 (8.2%)	0.696
**Headache**	7 (14.3%)	6 (12.2%)	0.766	0 (0)	0 (0)	1.000
**Skin capillary hyperplasia**	9 (18.4%)	1 (2.0%)	**0.033**	2 (4.1%)	0 (0)	0.155
**Hypothyroidism**	11 (22.4%)	1 (2.0%)	**0.002**	0 (0)	0 (0)	1.000
**Pneumonia**	1 (2.0%)	0 (0.0%)	0.317	0 (0)	0 (0)	1.000

PLR, platelet-to-lymphocyte ratio.

The bold values means P < 0.05, which is considered statistically significant.

There was no statistical difference in the incidence of most AEs between the two groups. However, the incidence of some immunotherapy-related adverse events (irAEs) of any grade in the low PLR group was significantly higher than that in the high PLR group, with no statistical difference in grade 3 or 4 AEs (**Table 4**).

## Discussion

It is known that the prognosis of patients with uHCC is poor due to drug resistance, frequent recurrence, and metastasis [32]. With the advent of immunomodulatory antibodies and molecular-targeted drugs, a new combination strategy combining TACE + TKIs + ICIs has shown favorable results for uHCC patients (17, 19). However, given that the biological heterogeneity of uHCC and the tumor microenvironment might impair treatment effectiveness, not all patients can benefit from this treatment and the high medical cost is also a worrisome issue. Therefore, it is warranted to identify the patients who are most likely to benefit from this triple therapy.

Accumulating evidence indicates that the inflammatory tumor microenvironment contributes to tumor occurrence and progression and may affect the prognosis of patients with malignancies (33, 34). PLR, as a biomarker that correlates systemic inflammation and immune function, has been shown to be a prognostic factor in various tumors (Chen et al., 2020; 35; 36). In the present study, we evaluated the prognostic value of pretreatment PLR for uHCC patients treated with TACE + TKIs + ICIs.

Our results suggested that patients with low pretreatment PLR had better prognosis than those with high pretreatment PLR. The mOS increased from 16.1 to 25.7 months (P < 0.001), and the corresponding median PFS increased from 10.2 to 14.9 months (P < 0.001). This indicated that pretreatment PLR grading could predict the survival outcomes of uHCC patients treated with TACE + TKIs + ICIs.

Univariate and multivariate Cox proportional hazards regression analysis showed that ALT, tumor size, and PLR were independent risk factors for OS and that tumor size, AFP (≥400 ug/L) and PLR were predictors for PFS. The results suggested that patients with larger tumors had a higher risk of all-cause mortality and tumor progression than those with smaller ones. It may be accounted for that the larger HCC generally has significant necrosis and inflammation pathophysiologically, which contribute to carcinogenesis and tumor progression (33, 37). What’s more, the larger HCCs have poorer response to TACE than smaller ones (38).

Immunotherapy related hepatotoxicity often presents as an increase in ALT or AST (39). Our results suggested that elevated ALT levels before TACE + TKIs + ICIs could predict the OS of uHCC patients, which was consistent with previous research results (40). Therefore, it is challenging for clinicians to manage the patients’ liver function. AFP is one of the biomarkers of HCC, and we found that elevated AFP levels were correlated with tumor progression. It indicated that AFP could be used as a potential biomarker to predict the tumor progression in uHCC patients treated with TACE + TKIs + ICIs. In addition, our results showed that patients with low pretreatment PLR had lower risks for tumor progression and all-cause mortality than those with high PLR, indicating that PLR is a promising biomarker to predict the survival outcomes of uHCC patients treated with TACE + TKIs + ICIs.

In reference to AEs, this study suggested that TACE + TKIs + ICIs was well-tolerated and its side effects were manageable. The incidences of some irAEs including skin capillary hyperplasia and hypothyroidism were significantly higher in the low PLR group than those in the high PLR group (P = 0.033, P = 0.002, respectively). It might be accounted for the stronger antitumor immune response in the low PLR group. And this result indicated that a low pretreatment PLR might be a predictor of the occurrence of irAEs. As the triple therapy may elicit strong immune responses, these irAEs should be carefully supervised in clinical practice.

While our study showed that the combination therapy of TACE + TKIs + ICIs was a promising approach to treat uHCC, it was also significant to delve into the potential role of second-line immunotherapy in uHCC if patients’ responses to the combination therapy were inadequate or the disease progressed. Some studies (41, 42) had investigated the efficacy of second-line immunotherapy, such as pembrolizumab and nivolumab, in advanced HCC and showed favorable results. However, given the variability in treatment response observed in immunotherapy, it is crucial to understand the responses to second-line treatments to optimize treatment selection and sequencing (43). In addition, it is also warranted to identify predictive biomarkers to aid in stratifying patients who are most likely to benefit from second-line immunotherapy.

Our study had some limitations. First, it was a retrospective and single-center study, which might cause selection bias. Second, the number of patients enrolled in this study was limited. Third, the cut-off value of PLR in our study was determined by the ROC curve analysis, which might not be representative. Therefore, further randomized case-controlled trials with a larger sample size are demanded to validate our findings.

## Conclusion

Our study suggested that the low pretreatment PLR (PLR ≤ 98.89) was an independent protective factor for the prognosis with uHCC patients treated with TACE + TKIs + ICIs. What’s more, the lower pretreatment PLR might also be an indicator of the occurrence of irAEs. Considering that PLR is an easily accessible indicator in clinical practice, it was helpful for clinicians to predict the prognosis and identify the patients with uHCC who were most likely to benefit from TACE + TKIs + ICIs.

## Data availability statement

The raw data supporting the conclusions of this article will be made available by the authors, without undue reservation.

## Ethics statement

The studies involving humans were approved by The Institutional Review Board of Wuhan Union Hospital. The studies were conducted in accordance with the local legislation and institutional requirements. The ethics committee/institutional review board waived the requirement of written informed consent for participation from the participants or the participants’ legal guardians/next of kin because this study was a retrospective study.

## Author contributions

YG: Investigation, Writing – original draft. WW: Investigation, Writing – review & editing. BS: Investigation, Writing – review & editing. TG: Funding acquisition, Writing – review & editing. KS: Visualization, Writing – review & editing. CZ: Conceptualization, Supervision, Writing – review & editing. XL: Conceptualization, Supervision, Writing – review & editing.
